# ﻿Two new synonyms in the subfamily Theridiinae (Araneae, Theridiidae)

**DOI:** 10.3897/zookeys.1120.90509

**Published:** 2022-09-05

**Authors:** Rui Zhong, Jie Liu, Yang Zhu

**Affiliations:** 1 Hubeiate Key Laboratory of Regional Development and Environmental Response, Faculty of Resources and Environmental Science, Hubei University, Wuhan 430062, Hubei, China; 2 State Key Laboratory of Biocatalysis and Enzyme Engineering, School of Life Sciences, Hubei University, Wuhan 430062, Hubei, China; 3 School of Nuclear Technology and Chemistry and Biology, Hubei University of Science and Technology, Xianning 437100, Hubei, China; 4 Wuhan Lvjia Technology Co., Ltd, Wuhan 430114, Hubei, China

**Keywords:** China, revision, taxonomy, *Theridion*

## Abstract

Two new synonyms in the subfamily Theridiinae Sundevall, 1833 (Araneae, Theridiidae) are reported. *Theridionhui* Zhu, 1998 is proposed as a junior synonym of *Theridioninnocuum* Thorell, 1875, and *Theridionqingzangense* Hu, 2001 is proposed as a junior synonym of *Phyllonetaimpressa* (L. Koch, 1881). Photos of habitus and copulatory organs are provided for both species.

## ﻿Introduction

The subfamily Theridiinae Sundevall, 1833, comprising all non-Hadrotarsinae genera without a trace of a colulus or colular setae, is the largest subfamily of Theridiidae (Liu et al. 2016). As the most speciose genus of this subfamily, *Theridion* Walckenaer, 1805 is in a particularly poor taxonomic state (Arnedo et al. 2007). More than two hundred species that are currently considered within the genus have not been revised since their preliminary description, most of them are lacking detailed descriptions and illustrations (Levy and Amitai 1982; Simon 1909; Levi 1957, 1963; Marples 1955; di Caporiacco 1934). More and more neglected morphological features have been noticed and given greater taxonomic value with the advancement of identification methods in the last few years (Agnarsson 2004). As a result, many species or small species groups have been separated from *Theridion* (Wunderlich 2008; Knoflach et al. 2009; Yoshida 2001). However, the genus remains the most speciose genus of Theridiidae, with 585 valid species, nearly a quarter of all known theridiids (World Spider Catalog 2022). In the current paper, two new synonyms in *Theridion* are proposed: *T.hui* as a junior synonym of *T.innocuum*, and *T.qingzangensis* as a junior synonym of *Phyllonetaimpressa*.

## ﻿Materials and methods

All specimens are kept in 100% ethanol and examined with an Olympus SZX16 stereomicroscope. Details were further investigated with an Olympus BX51 compound microscope. The materials examined are deposited in Centre for Behavioral Ecology and Evolution, College of Life Sciences, Hubei University, Wuhan, China.

### ﻿Abbreviations used in the figures

**CO** copulatory opening;

**CD** copulatory duct;

**FD** fertilization duct;

**ST** subtegulum;

**S** spermatheca;

**C** conductor;

**E** embolus;

**T** tegulum.

## ﻿Taxonomy

### ﻿Family Theridiidae Sundevall, 1833

**Subfamily Theridiinae Sundevall, 1833**

#### Genus *Theridion* Walckenaer, 1805

##### ﻿ Theridion innocuum

Taxon classificationAnimaliaAraneaeTheridiidae

Thorell, 1875

30173808-1E05-5C98-A9C9-CBAD9016908D

Figs 1 , 4

Theridion innocuum Thorell 1875a: 65 (description of male); Thorell 1875b: 50 (description of male); Azheganova 1968: 58, figs 102, 113 (male and female); Izmailova 1972: 43, fig. 1 (female); Izmailova 1989: 92, fig. 72 (female); Le Peru 2011: 478, fig. 824 (female); Esyunin and Stepina 2014: 49, figs 14, 15 (female); Zamani et al. 2021: 295, fig. 12F–N (male and female).
Theridion theridioides : Hu and Wu 1989: 137, figs 110.1, 110.2 (female, misidentified).
Theridion hui Zhu 1998: 150, fig. 91A–C (description of female); Song et al. 1999: 137, fig. 73I, J (female). New synonym.

###### Material examined.

**China, Xinjiang Uygur Autonomous Region**: Hemu Scenic Area, Hemuhanas Mongolian Township, Burqin County, Altay Region, 48°34'28"N, 87°26'23"E, 1445 m, 1 July 2021, Chen Jian and Liu Jie leg.: 1 female.

**Figure 1.**
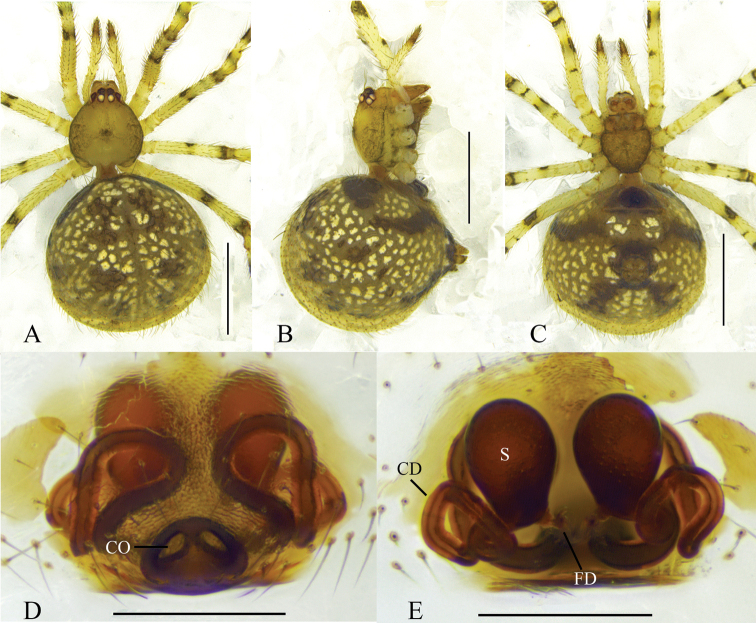
*Theridioninnocuum* Thorell, 1875 **A–C** female habitus (**A** dorsal **B** lateral **C** ventral) **D** epigyne, ventral **E** vulva, dorsal. Scale bars: 1 mm (**A–C**); 0.2 mm (**D, E**). CO = copulatory opening; CD = copulatory duct; FD = fertilization duct; S = spermatheca.

###### Description.

See Azheganova (1968) and Thorell (1875a, 1875b).

###### Justification of the synonymy.

*Theridionhui* was first described by Zhu (1998), based on a female collected in Xinjiang Uygur Autonomous Region, China and previously identified as *Theridiontheridioides* by Hu and Wu (1989). The female holotype of *T.hui* bears all the diagnostic features of *T.innocuum*, including the messy dark brown spots on abdomen, peach-shaped fossa of the epigyne and thick, long, similarly twisted copulatory ducts. The original illustrations of the epigyne of *T.hui* perfectly match the coloured photos of the same provided for *T.innocuum* by Zamani et al. (2021) (compare Zhu 1998: fig. 91A–C with Zamani et al. 2021: fig. 12K–N). Although the types of *T.innocuum* and *T.hui* were unavailable for examination, our comparison based on a female specimen collected in Xinjiang Uygur Autonomous Region and the illustrations and descriptions provided by Zamani et al. (2021) and Zhu (1998) enabled us to confidently consider *T.hui* as a junior synonym of *T.innocuum*.

###### Distribution.

Ukraine, Russia (Europe to South Siberia), Iran, Kazakhstan, China (new record, Fig. 4).

#### Genus *Phylloneta* Archer, 1950

##### ﻿ Phylloneta impressa

Taxon classificationAnimaliaAraneaeTheridiidae

(L. Koch, 1881)

2B3D4AFB-D3E4-57A2-B7C7-FF1811673B71

Figs 2 , 3 , 4

Steatoda sisyphia Menge 1868: 161, pl. 30, fig. 69 (male and female, misidentified per Simon 1914: 295).
Theridion sisyphum Simon 1881: 100 (partly misidentified per Simon 1914: 295).
Theridion impressum L. Koch 1881: 45, pl. 2, fig. 1 (description of male).
Theridion impressum intermedium Kulczyński 1885: 27 (description of female); Chyzer and Kulczyński 1894: 33, pl. 1, fig. 26 (male and description of female); Bösenberg 1902: 99, pl. 9, fig. 111 (male and female); Fedotov 1912: 61, fig. 1 (male); Simon 1914: 257, 295, figs 509, 512–513 (male and female).
Theridion cornutum Yurinich and Drensky 1917: 116, 136, pl. 1, figs 1–3 (description of juvenile; preoccupied by Wider 1834).
Theridion botezati Roșca 1935: 243, fig. 3 (description of male and female); Roșca 1936: 198, fig. 5 (male and female).
Theridion impressum Wiehle 1937: 152, figs 81–84 (male and female).
Theridion cornutum Drensky 1939: 85, fig. 1a, b (female, synonym of Theridionbotezati).
Theridion impressum Nakatsudi 1942: 9, fig. 1C (female).
Theridion frigicola Chamberlin and Ivie 1947: 27, figs 14, 15 (description of male and female).
Allotheridion impressum Archer 1950: 20 (male and female transferred from Theridion).
Theridion impressum Locket and Millidge 1953: 67, fig. 44C, D (male and female); Levi 1957: 89, figs 321, 326–328 (male and female, synonym); Azheganova 1968: 58, figs 101, 112 (male and female); Tystshenko 1971: 150, figs 401, 426 (male and female); Miller 1971: 194, pl. 35 figs 4, 5 (male and female); Izmailova 1972: 44, fig. 2 (female); Palmgren 1974: 18, figs 4.1–4 (male and female); Punda 1975: 64, figs 137, 138 (female); Legotai and Sekerskaya 1982: 50, figs III.22, 24 (male and female); Müller 1982: 248, fig. 4 (female); Roberts 1985: 184, fig. 81e (male and female); Legotai and Sekerskaya 1989: 224, figs LXIX.22, 24 (male and female); Heimer and Nentwig 1991: 302, fig. 808 (male and female); Deltshev 1992: 17 (synonym); Roberts 1995: 282, fig. (male and female); Mcheidze 1997: 192, figs 388–391 (male and female); Bellmann 1997: 68, fig. (female); Zhu 1998: 161, fig. 100A–E (male and female); Roberts 1998: 296, fig. (male and female); Song et al. 1999: 138, fig. 74A–B, K–L (male and female); Hu 2001: 580, figs 393.1–4 (male and female); Almquist 2005: 99, fig. 121a–h (male and female); Paquin and Dupérré 2006: 27, figs 74–79 (male and female).
Phylloneta impressa Wunderlich 2008: 393, figs 554–557 (male).
Theridion impressum Le Peru 2011: 478, fig. 823 (male and female).
Phylloneta impressa Kaya and Ugurtas 2011: 148, figs 10–11 (male and female); Quasin and Uniyal 2012: 59, figs 1–2, 3a–c (female); Tilly and Tilly 2019: 8, fig. 7A, B (male); Vanuytven 2021: 43, 233, figs A.32c, B.244 (male).
Theridion qingzangensis : Hu 2001: 586, fig. 398.1–5 (description of female). New synonym.

###### Material examined.

**China**, Xinjiang Uygur Autonomous Region: State Road 217, Kaerjiao Town, Jimunai County, Altay Region,47°5'27"N, 86°36'46"E, 915 m, 30 June 2021, Chen Jian and Liu Jie leg.: 3 female; Guozigou – Sailimu Lake Scenic Spot, Ili Kazakh Autonomous Prefecture, 44°3'32"N, 80°52'40"E, 2101 m, 4 July 2021, Chen Jian and Liu Jie leg.: 3 male; Akeqi Animal Husbandry Village, Karasu Town, Zhaosu County, Ili Kazakh Autonomous Prefecture, 42°54'7"N, 80°53'37"E, 1810 m, 5 July 2021, Chen Jian and Liu Jie leg.: 1 female and 6 male; National Road 577, near Tasbulak Village, Zhaosu County, Ili Kazakh Autonomous Prefecture, 43°9'49"N, 81°8'15"E, 3178 m, 5 July 2021, Chen Jian and Liu Jie leg.: 15 female and 5 male; Duku Highway near the Gongnaisi River, Xinyuan County, Ili Kazakh Autonomous Prefecture, 43°16'36"N, 84°18'0"E, 1680 m, 6 July 2021, Chen Jian and Liu Jie leg.: 1 male; Provincial Road 315, Keling Township, Nileke County, Ili Kazakh Autonomous Prefecture, 43°50'38"N, 82°22'10"E, 1807 m, 6 July 2021, Chen Jian and Liu Jie leg.: 2 female; National Road 218, near Yinggar Village, Xinyuan County, Ili Kazakh Autonomous Prefecture, 43°14'8"N, 82°18'1"E, 1966 m, 7 July 2021, Chen Jian and Liu Jie leg.: 3 female; 135 Township Road, near Changyuan Gas CNG Gas Station, Kuqa City, Aksu Region, 41°44'24"N, 83°7'44"E, 1057 m, 8 July 2021, Chen Jian and Liu Jie leg.: 4 female; National Road 218, near Bosten Lake Scenic Area, Bohu County, Bayingoleng Mongolia Autonomous Prefecture, 41°48'22"N, 86°21'51"E, 1077 m, 9 July 2021, Chen Jian and Liu Jie leg.: 14 female and 1 male; Tianshan Tianchi Scenic Area, No. 501, Junggar Road, Fukang City, Changji Prefecture, 43°55'11"N, 88°7'30"E, 1910 m, 12 July 2021, Chen Jian and Liu Jie leg.: 6 female.

###### Description.

See Paquin and Dupérré (2006), Kaya and Uğurtaş (2011), and Quasin and Uniyal (2012).

###### Justification of the synonymy.

*Theridionqingzangense* was first described as a new species similar to *P.impressa*, based on a female collected in Qinghai-Tibet Plateau of China (Hu 2001). The most important diagnostic features of *T.qingzangense* were the oval spermathecae and long copulatory ducts that are initially thick but become thinner and twisted at the junction with the spermathecae. Comparison of the epigyne of *T.qingzangense* (Hu 2001: figs 398.1–5) with that of *P.impressa* (Hu 2001: figs 393.1–4; Wiehle 1937: figs 81, 83, 84; Quasin and Uniyal 2012: fig. 3a–c) suggests that *P.impressa* has all the diagnostic features of *T.qingzangense*. In addition, we collected specimens of both sexes in Xinjiang Uygur Autonomous Region, China (Fig. 4), and the female perfectly matches the illustrations provided for *T.qingzangense*, and the male matches the illustrations available for *P.impressa*. Though the copulatory ducts from a few of our specimens show slight variations, these differences, which we consider as intraspecific variation, are minor and do not provide enough evidence for erection of a separate species. Thus, *T.qingzangense* is hereby considered as a junior synonym of *P.impressa*.

**Figure 2.**
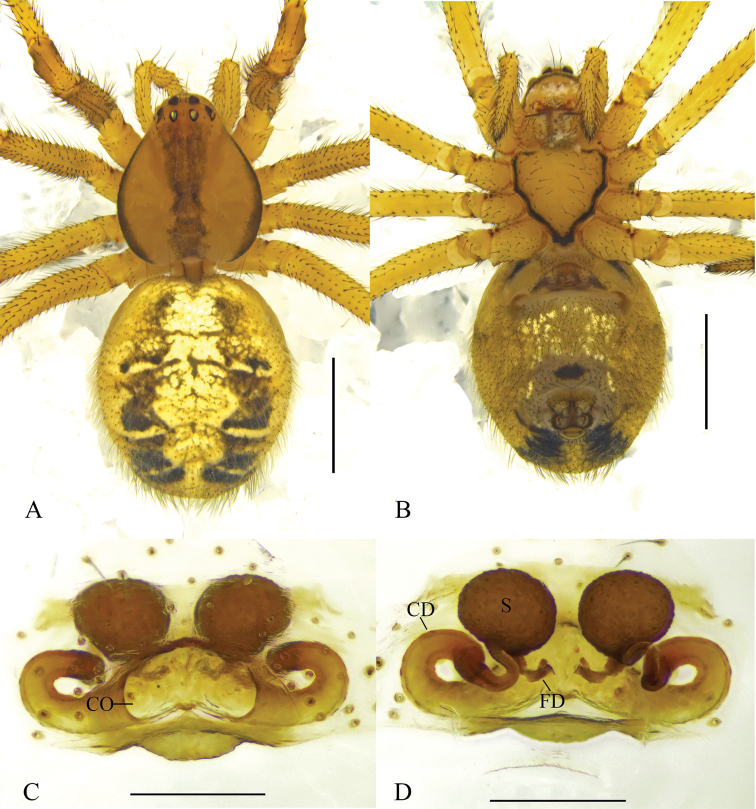
*Phyllonetaimpressa* (L. Koch, 1881) **A, B** female habitus (**A** dorsal **B** ventral) **C** epigyne, ventral **D** vulva, dorsal. Scale bars: 1 mm (**A, B**); 0.2 mm (**C, D**). CO = copulatory opening; CD = copulatory duct; FD = fertilization duct; S = spermatheca.

###### Distribution.

North America, Europe, Turkey, Caucasus, Russia (Europe to Far East), Kazakhstan, Iran, Central Asia, China (Fig. 4), India.

**Figure 3.**
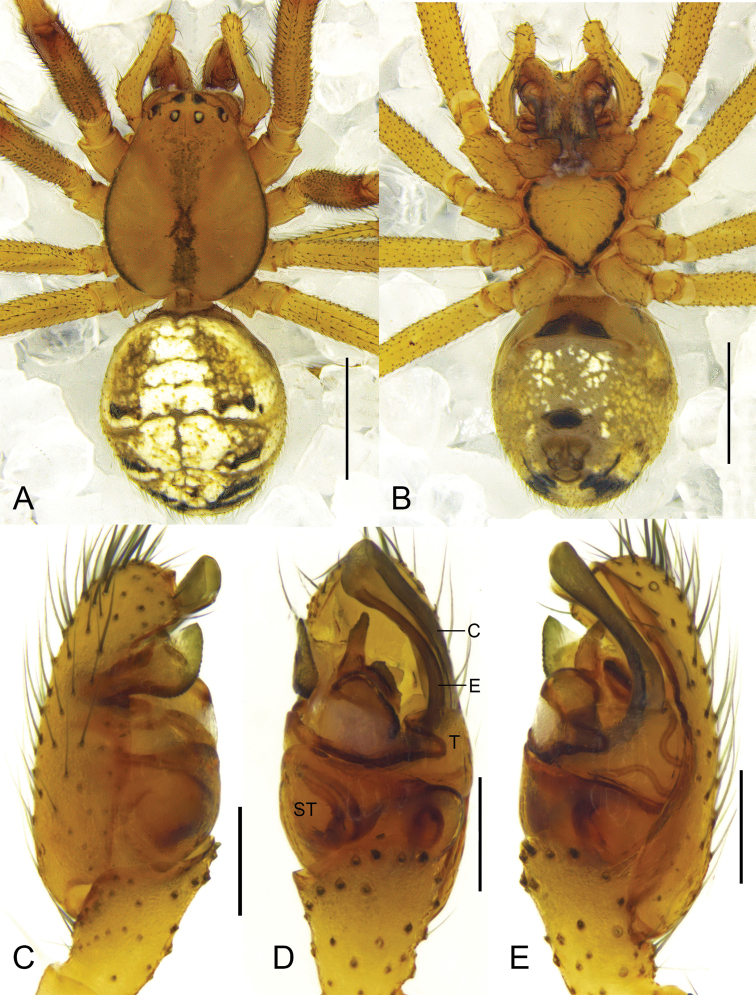
*Phyllonetaimpressa* (L. Koch, 1881) **A, B** male habitus (**A** dorsal **B** ventral) **C–E** left male palp (**C** prolateral **D** ventral **E** retrolateral). Scale bars: 1 mm (**A, B**); 0.2 mm (**C–E**). ST = subtegulum; C = conductor; E = embolus; T = tegulum.

**Figure 4.**
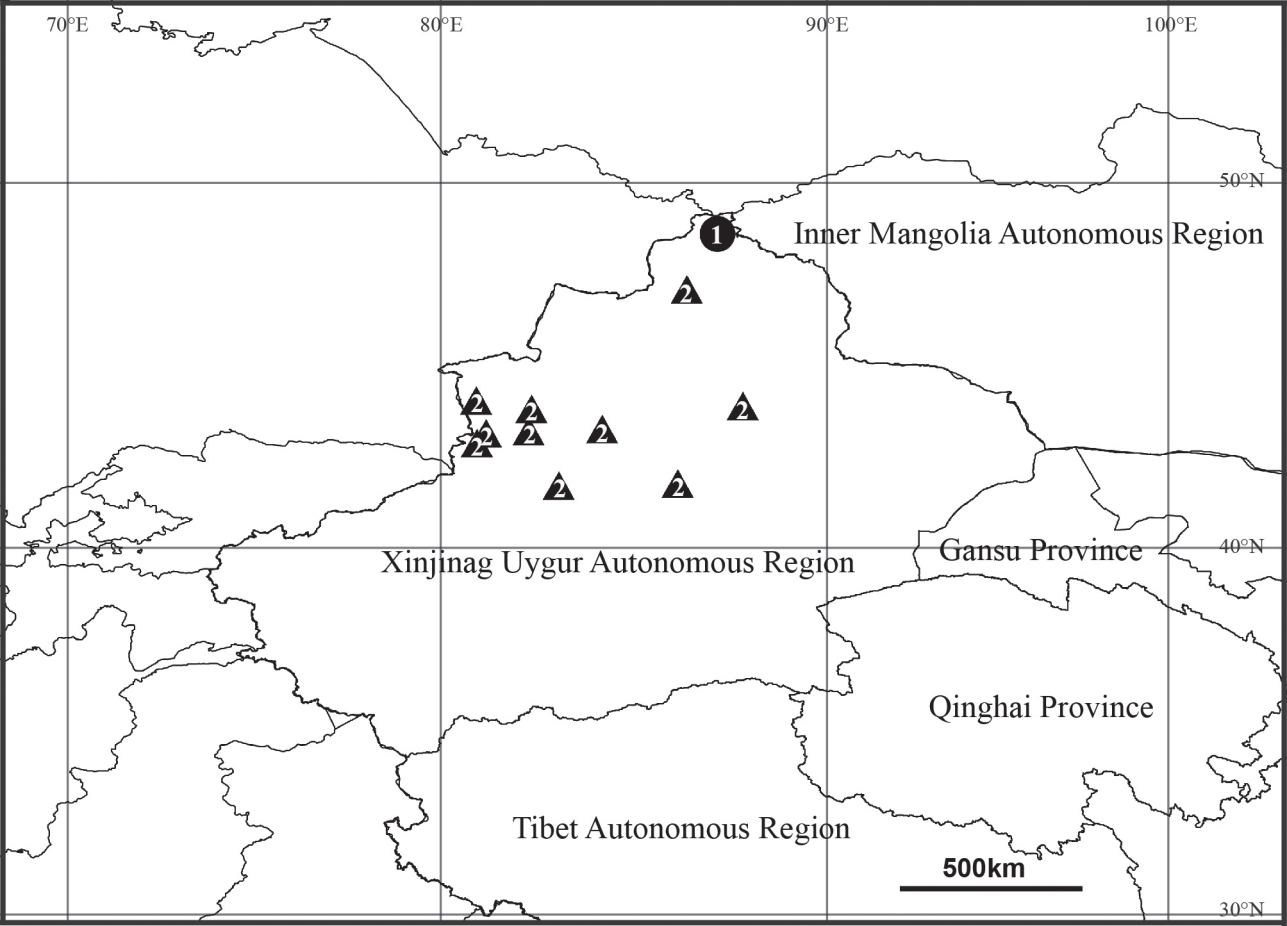
Collection localities in the Xinjiang Uygur Autonomous Region, China: **1***T.innocuum***2***P.impressa*.

## Supplementary Material

XML Treatment for Theridion innocuum

XML Treatment for Phylloneta impressa
